# Supplementary material for: Antimicrobial Resistance and Comparative Genome Analysis of Klebsiella pneumoniae Strains Isolated in Egypt
Source: Microorganisms. 2021 Sep 5;9(9):1880. doi: 10.3390/microorganisms9091880 (PMC8465295; doi:10.3390/microorganisms9091880)
Supplement: Supplementary file 1 [file microorganisms-09-01880-s001.zip › microorganisms-1371655-supplementary.pdf]

Table S1. Antibiotic resistance gene analysis of Egyptian *Klebsiella pneumoniae* strains SF, SK and HM.

	**SF**	**SK**	**HM**
Acquired antimicrobial resistance genes [a,b]	*aac*(6')-Il, *aadA22*, *aadA23*,<br><br>*aph*(3'')-Ib, *aph*(3')-VIb, *aph*(6)-Id<br><br>bla_{SHV-28}, bla_{VIM-29}, bla_{CMY-4}, $bla_{CTX-M-14b}$<br><br>*dfrA1, floR, fosA, oqxA, oqxB, sul2, tet(A)*	*aac*(6')-Il, *aadA22*, *aadA23*, ***aac*(6')-Ib**, ***aac*(6')-Ib-cr,** *aph*(3'')-Ib, *aph*(3')-VIb, *aph*(6)-Id<br><br>bla_{SHV-28}, bla_{VIM-29}, bla_{CMY-4}, $bla_{CTX-M-14b}$, $\mathbf{bla_{OXA-9}}$<br><br>*dfrA1, floR, fosA, oqxA, oqxB, sul2, tet(A)*	*aac*(6')-Il, *aadA22*, *aadA23*, ***aac*(6')-Ib, *aac*(6')-Ib-cr,** *aph*(3'')-Ib, *aph*(3')-VIb, *aph*(6)-Id<br><br>bla_{SHV-28}, bla_{VIM-29}, bla_{CMY-4}, $bla_{CTX-M-14b}$, $\mathbf{bla_{OXA-9}}$<br><br>*dfrA1, floR, fosA, oqxA, oqxB, sul2, tet(A)*
Chromosomal point mutations associated with antimicrobial resistance [a]	***ompK36***: N49S, L59V, L191S, F207W, A217S, N218H, D224E, L228V, E232R, T254S<br><br>***ompK37:*** I70M, I128M<br><br>***acrR***: P161R, G164A, F172S, R173G, L195V, F197I, K201M	***ompK36***: N49S, L59V, L191S, F207W, A217S, N218H, D224E, L228V, E232R, T254S<br><br>***ompK37***: I70M, I128M<br><br>***acrR:*** P161R, G164A, F172S, R173G, L195V, F197I, K201M	***ompK36:*** N49S, L59V, L191S, F207W, A217S, N218H, 224E, L228V, E232R, T254S<br><br>***ompK37***: I70M, I128M<br><br>***acrR:*** P161R, G164A, F172S, R173G, L195V, F197I, K201M

[a] Acquired antimicrobial resistance genes and chromosomal point mutations were identified using ResFinder 3.2 (Center for Genomic Epidemiology http://www.genomicepidemiology.org/) [1].
[b] Differences in the acquired antimicrobial resistance genes carried by *K. pneumoniae* strains SK and HM that are not present in strain SF are in bold.

Supplementary Figure legends

Figure S1. Comparison of draft genome of *Klebsiella pneumoniae* strain SK with those of *K. pneumoniae* strains HM and SF. (**A**) The panel shows the comparison of the *K. pneumoniae* SK draft genome with the genomes of HM and SF using GCview (http://stothard.afns.ualberta.ca/cgview_server/) [2]. The outer three rings display the genes (CDS, coding sequences) and contigs of the *K. pneumoniae* SK draft genome [3]. The blue and green shaded rings illustrate the BLAST results when the genome sequences of strains HM and SF, respectively, are compared to the SK genome. Shaded regions indicate homology and major differences (*i.e.* gaps between *K. pneumoniae* SK and HM) are highlighted by a red arc. (**B**) The panel shows a higher magnification image of the red arc in A). Genes and features present in the genome of strain SK, but absent from SF, are labelled. Insertion sequences were identified by ISfinder (https://www-is.biotoul.fr/blast/resultat.php) [4].

Figure S2. Analysis of the IncFII(K) plasmid carried by *K. pneumoniae* strain KPE16. (**A**) The panel shows the comparison of the *K. pneumoniae* plasmid p2 (CP009115.1) [5] with the KPE16 draft genome and various KPE16 contigs, using GCview [2]. The outer two rings display the genes on p2 (CDS), with plasmid replicons and selected features labelled. The blue, green, gold and red shading illustrates the BLAST results when the draft genome sequence of KPE16 and KPE16 contigs 27, 30 and 35, respectively, are compared with p2. Shaded regions indicate homology. (**B**) The panel shows the alignment of *K. pneumoniae* plasmid p2 (CP009115.1) [5] with KPE16 contigs 30 and 35, using the Artemis Comparison Tool (ACT) [6]. The alignment is shown by red and blue banding. Blue banding indicates that the sequences are inverted with respect to each other. Selected features that are shared between sequences are indicated.

Figure S3. Comparison of the *K. pneumoniae* KPE16 draft genome with those of *K. pneumoniae* SF, SK, HM and SP. The figure shows the comparison of the *K. pneumoniae* KPE16 draft genome with those of SF, SK, HM and SP, using GCview [2]. The outer two rings display KPE16 genes (CDS) on both strands and the third ring shows the contigs of the *K. pneumoniae* KPE16 draft genome. The blue, green, gold and red rings illustrate the BLAST results when the genome sequences of strains SF, SK, HM and SP, respectively, are compared to the KPE16 genome. Shaded regions indicate homology and major differences are gaps. *K. pneumoniae* KPE16 contig 19 is highlighted as this sequence is absent from the SF, SK, HM and SP genomes.

Figure S4. Comparison of plasmid pKP1-19 with the *K. pneumoniae* KPE16 draft genome. (**A**) The panel shows the comparison of *K. pneumoniae* plasmid pKP1-19 (CP012884.1) [7] with the complete genome and contig 19 from strain KPE16, using GCview [2]. The outer two rings display the genes on pKP1-19

(CDS), with plasmid replicons and selected features labelled. The blue and green rings illustrate the BLAST results when the draft genome sequence of KPE16 and KPE16 contig 19, respectively, are compared with pKP1-19. Shaded regions indicate homology. (**B**) The panel shows the alignment of *K. pneumoniae* plasmid pKP1-19 (CP012884.1) [7] with KPE16 contig 19, using ACT [6]. The alignment is shown by blue banding, which indicates that the sequences are inverted with respect to each other.

Figure S5. Analysis of IncQ1 replicons carried by Egyptian *K. pneumoniae* and EAEC strains. (**A**) The panel shows a comparison of plasmid pRSF1010 (M28829) [8] with various contigs from Egyptian *K. pneumoniae* strains and EAEC strain E36 [9], using GCview [2]. The outer two rings display the genes on pRSF1010 (CDS), with the IncQ1 plasmid replicon labelled. The blue, green and gold shading illustrates the BLAST results when contig 58 from *K. pneumoniae* SP, contig 55 from EAEC E36 [9] and contig 42 from *K. pneumoniae* KPE16, respectively, are compared with pRSF1010. Shaded regions indicate homology. (**B**) The panel shows the alignment of plasmid pRSF1010 (M28829) [8] with contig 58 from *K. pneumoniae* SP, contig 42 from *K. pneumoniae* KPE16 and contig 55 from EAEC E36 [9], using ACT [6]. The aligned sequences are shown by red banding, which corresponds to the IncQ1 *repA* and *sul2*, *aph*(3'')-Ib and *aph*(6)-Id antibiotic resistance genes [10]. Note that contig 42 from *K. pneumoniae* KPE16 carries an addition *aph*(3')-Ia resistance gene.

Figure S6. Analysis of the IncM replicon carried by *K. pneumoniae* strain SK. (**A**) The panel shows a comparison of plasmid pKpn14-4 (CP047704.1) [11] with the complete genome and contig 38 of *K. pneumoniae* strain SK, using GCview [2]. The outer two rings display the genes on pKpn14-4 (CDS), with the IncM replicon and $bla_{\text{CTX-M-14b}}$ ESBL gene labelled. The blue and green shading illustrates the BLAST results when the complete genome and contig 38 from *K. pneumoniae* SK, respectively, are compared with pKpn14-4. Shaded regions indicate homology. (**B**) The panel shows the alignment of plasmid pKpn14-4 (CP047704.1) [11] with contig 38 from *K. pneumoniae* SK, using ACT [6]. The aligned sequences are shown by red banding.

Figure S7. Analysis of proteins implicated in colistin resistance in *K. pneumoniae*. The figure show the alignment of the (**A**) PhoP, (**B**) PhoQ, (**C**) PmrA, (**D**) PmrB, (**E**) MgrB, (**F**) YciB/ LapB and (**G**) LpxM/ MsbB proteins found in *K. pneumoniae* strains MGH 78578, KPE16, SF, SK, HM and SP. In all panels, differences from the colistin sensitive *K. pneumoniae* MGH 78578 proteins are highlighted red.

Supplementary References

[1] E. Zankari, H. Hasman, S. Cosentino, M. Vestergaard, S. Rasmussen, O. Lund, et al., Identification of acquired antimicrobial resistance genes, J Antimicrob Chemother. 67 (2012) 2640-4.
[2] J.R. Grant, P. Stothard, The CGView Server: a comparative genomics tool for circular genomes, Nucleic Acids Res. 36 (2008) W181-4.
[3] H. Attia, R. Szubin, A.S. Yassin, J.M. Monk, R.K. Aziz, Draft Genome Sequences of Four Metallo-Beta-Lactamase-Producing Multidrug-Resistant *Klebsiella pneumoniae* Clinical Isolates, Including Two Colistin-Resistant Strains, from Cairo, Egypt, Microbiol Resour Announc. 8 (2019).
[4] Z. Zhang, S. Schwartz, L. Wagner, W. Miller, A greedy algorithm for aligning DNA sequences, J Comput Biol. 7 (2000) 203-14.
[5] D. van Duin, F. Perez, S.D. Rudin, E. Cober, J. Hanrahan, J. Ziegler, et al., Surveillance of carbapenem-resistant *Klebsiella pneumoniae*: tracking molecular epidemiology and outcomes through a regional network, Antimicrob Agents Chemother. 58 (2014) 4035-41.
[6] T.J. Carver, K.M. Rutherford, M. Berriman, M.A. Rajandream, B.G. Barrell, J. Parkhill, ACT: the Artemis Comparison Tool, Bioinformatics. 21 (2005) 3422-3.
[7] K.W. Lee, K. Arumugam, R.W. Purbojati, Q.X. Tay, R.B. Williams, S. Kjelleberg, et al., Draft Genome Sequence of *Klebsiella pneumoniae* Strain KP-1, Genome Announc. 1 (2013).
[8] P. Scholz, V. Haring, B. Wittmann-Liebold, K. Ashman, M. Bagdasarian, E. Scherzinger, Complete nucleotide sequence and gene organization of the broad-host-range plasmid RSF1010, Gene. 75 (1989) 271-88.
[9] R. Abdelwahab, M. Yasir, R.E. Godfrey, G.S. Christie, S.J. Element, F. Saville, et al., Antimicrobial resistance and gene regulation in Enteroaggregative *Escherichia coli* from Egyptian children with diarrhoea: Similarities and differences, Virulence. 12 (2021) 57-74.
[10] A. Carattoli, E. Zankari, A. Garcia-Fernandez, M. Voldby Larsen, O. Lund, L. Villa, et al., *In silico* detection and typing of plasmids using PlasmidFinder and plasmid multilocus sequence typing, Antimicrob Agents Chemother. 58 (2014) 3895-903.
[11] P. Kohler, N. Tijet, H.C. Kim, J. Johnstone, T. Edge, S.N. Patel, et al., Dissemination of Verona Integron-encoded Metallo-β-lactamase among clinical and environmental Enterobacteriaceae isolates in Ontario, Canada, Sci Rep. 10 (2020) 18580.

Supplementary Figure S1.

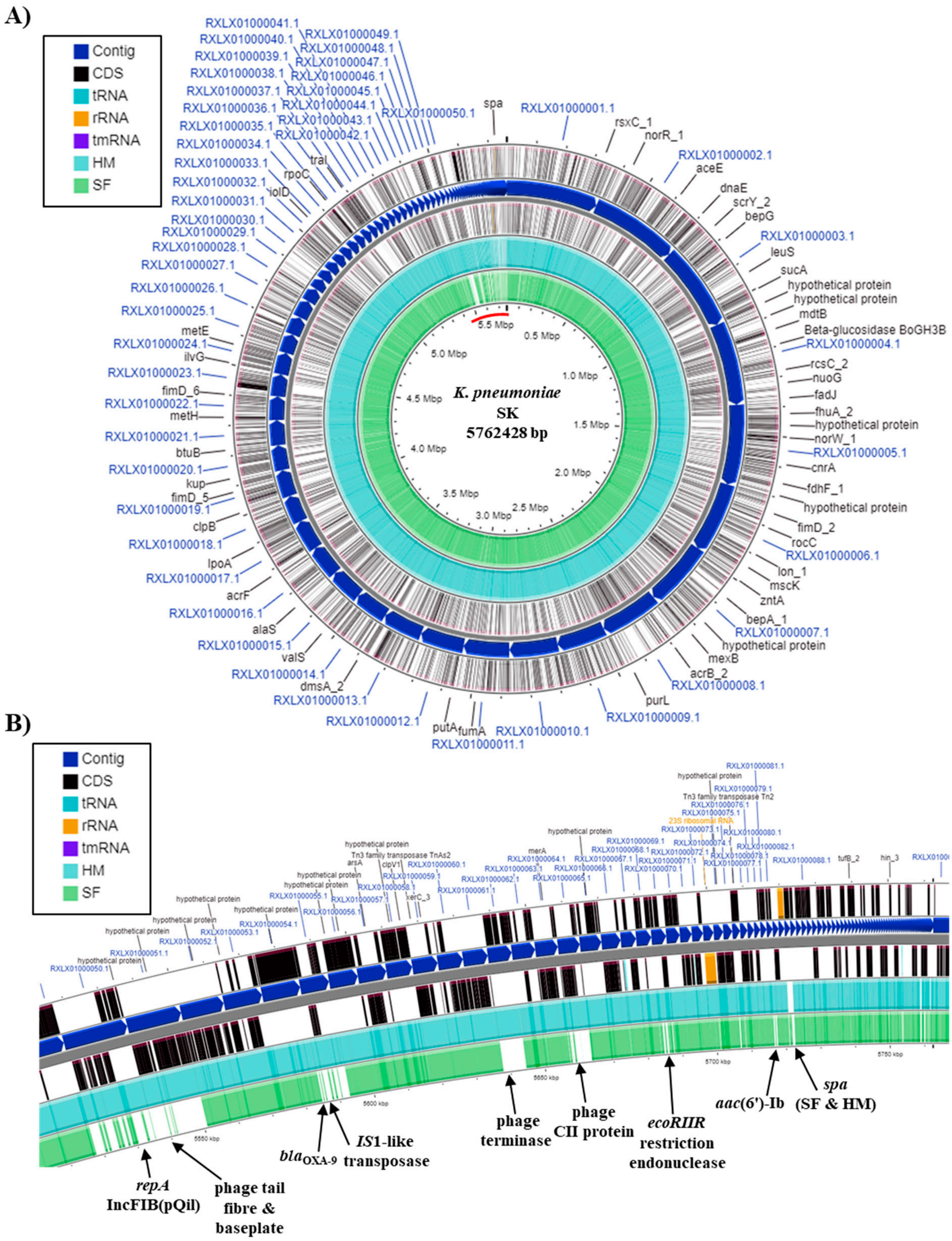

Supplementary Figure S2.

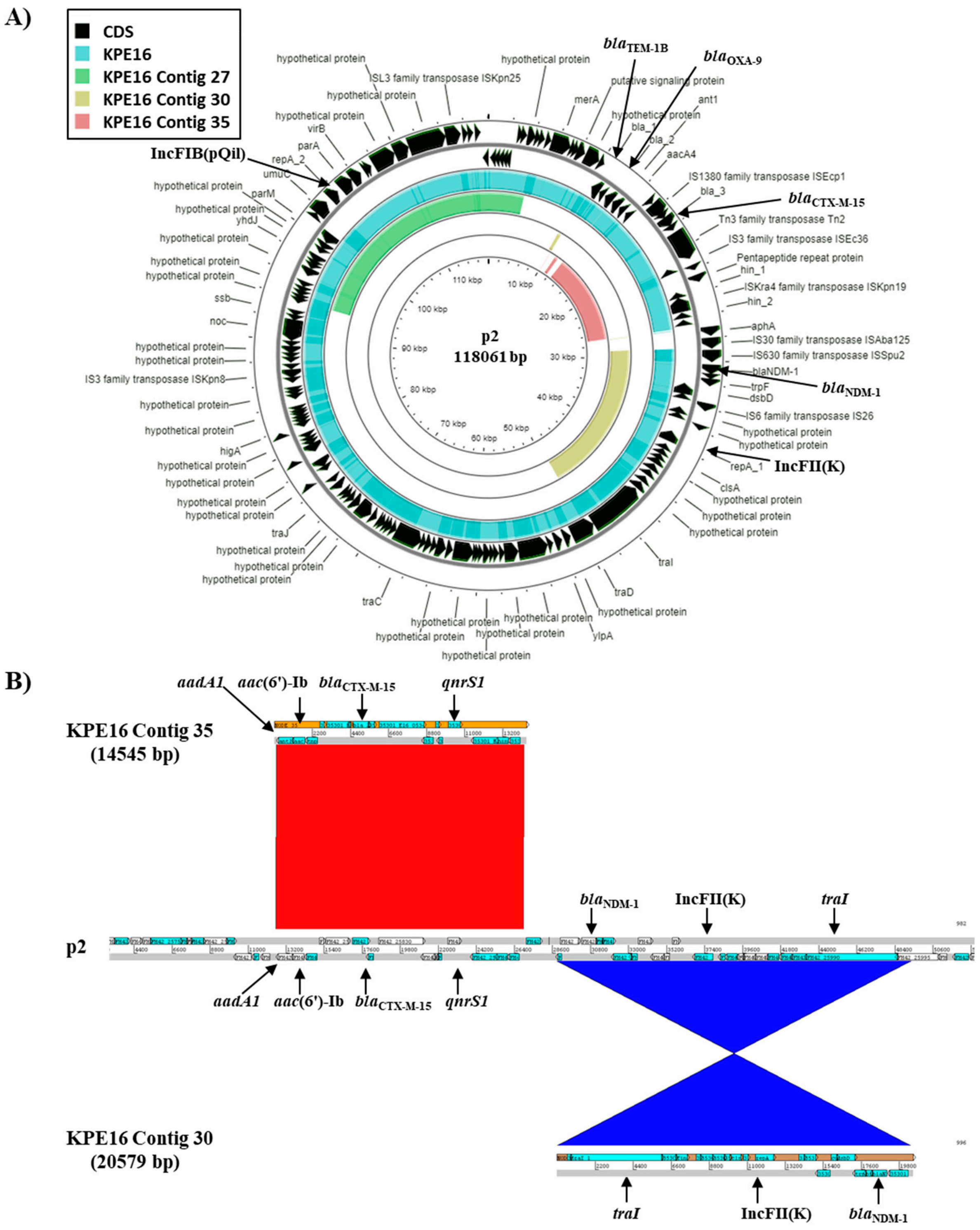

Supplementary Figure S3.

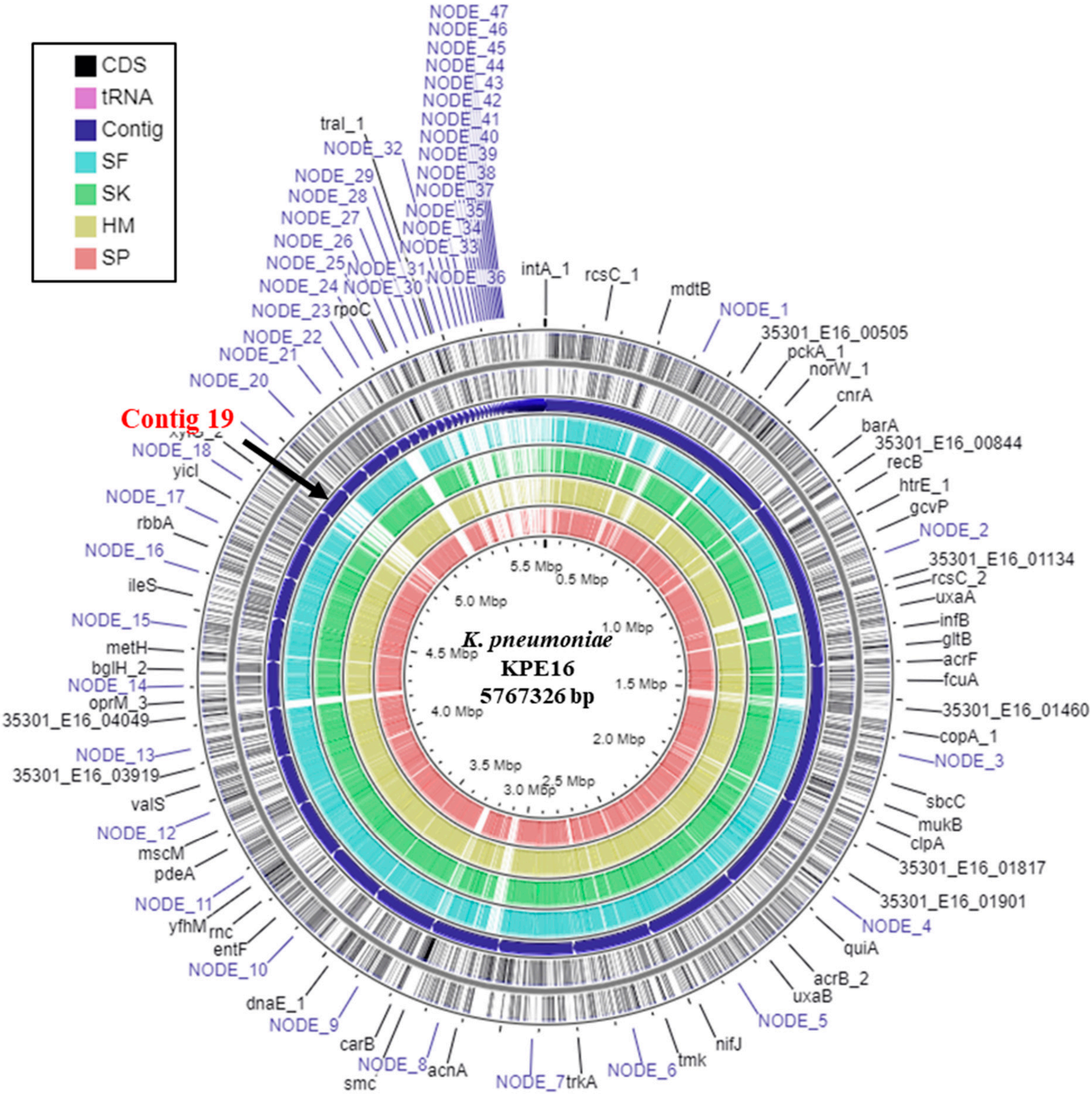

Supplementary Figure S4.

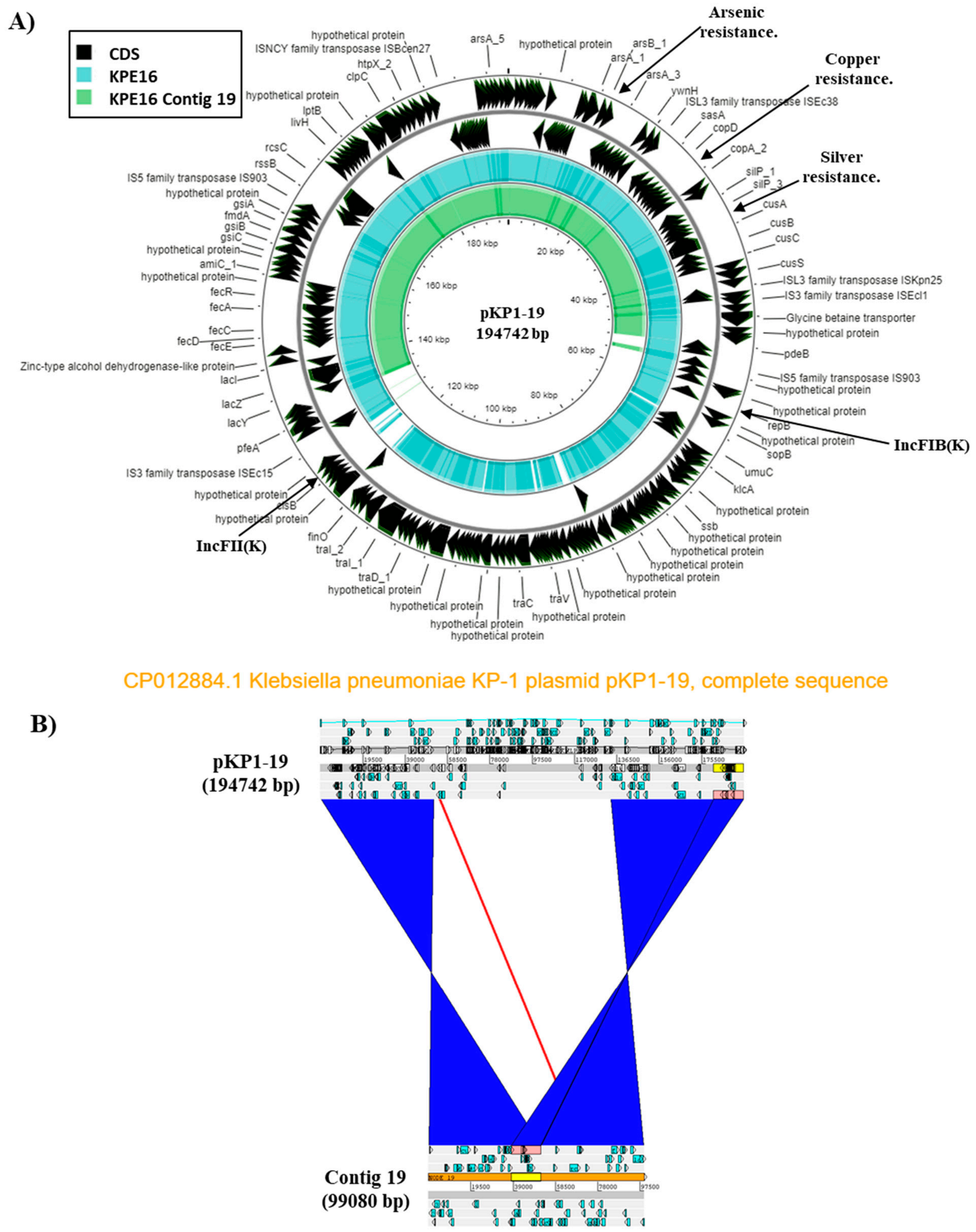

Supplementary Figure S5.

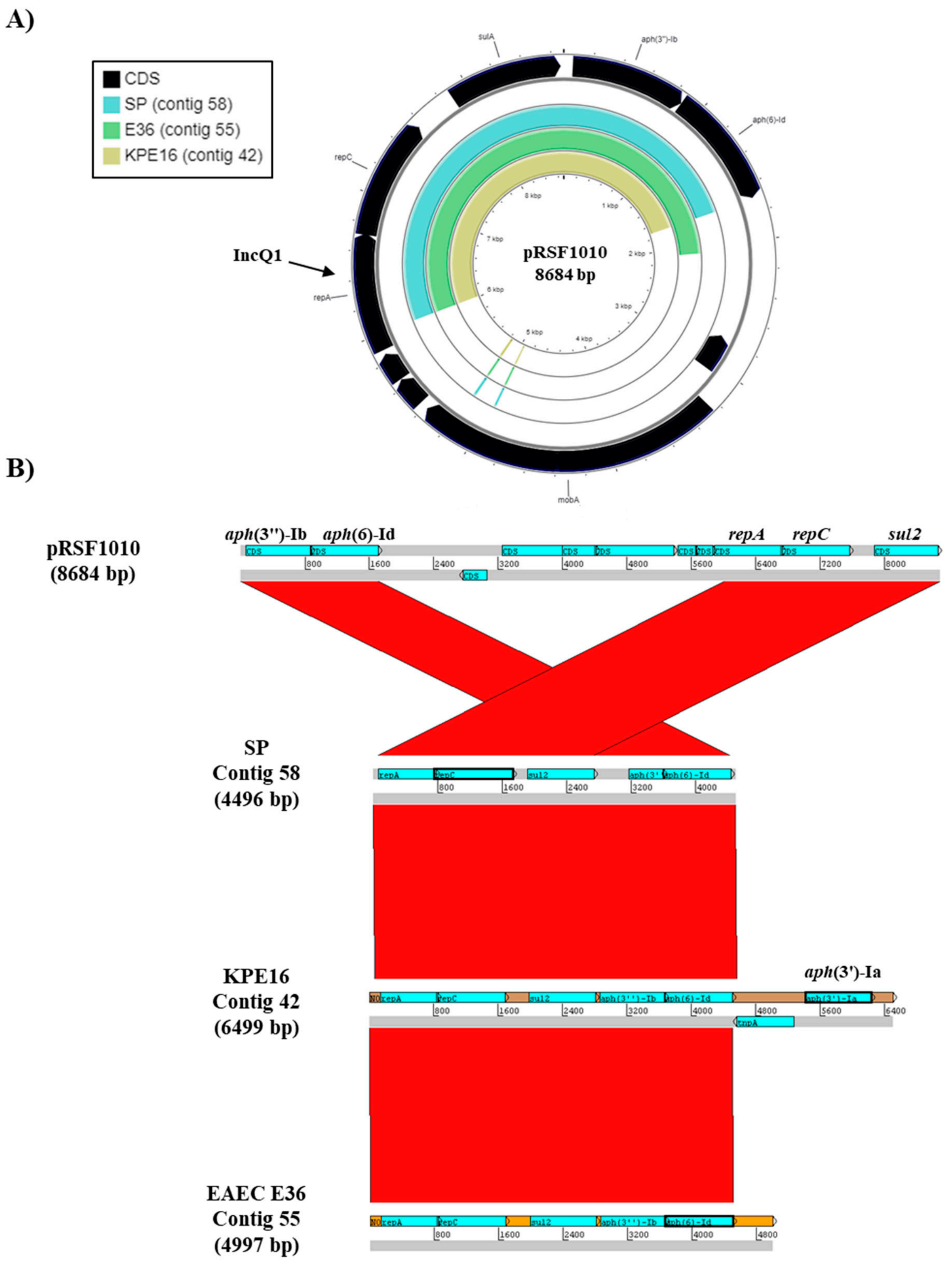

Supplementary Figure S6.

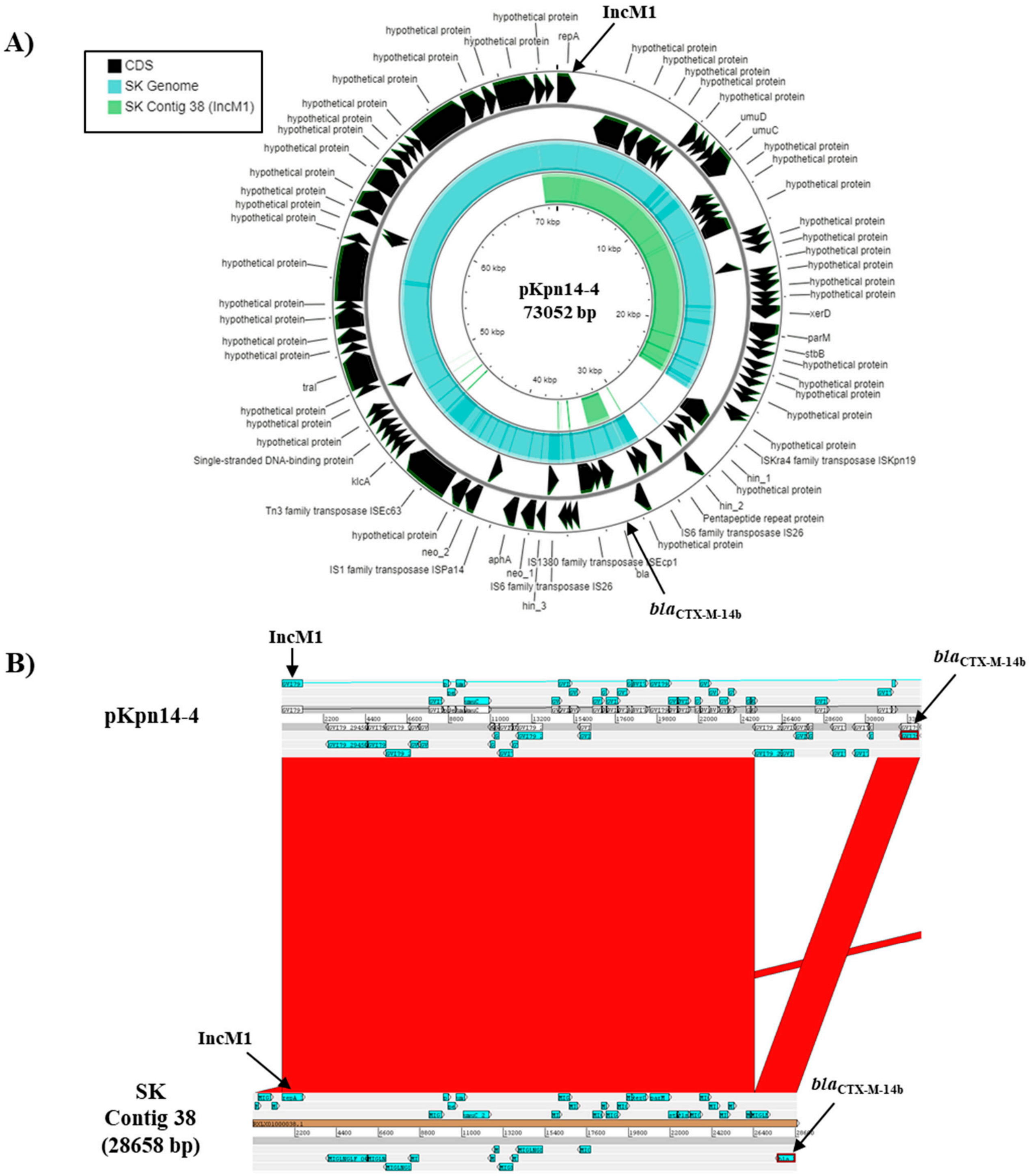

Supplementary Figure S7.

(A) **PhoP**

```
PhoP KPE16          MRVLVVEDNALLRHHLKVQLQELGHQVDAAEDAREADYYLGEHLPDIAIVDLGLPDEDGL
PhoP MGH78578       MRVLVVEDNALLRHHLKVQLQELGHQVDAAEDAREADYYLGEHLPDIAIVDLGLPDEDGL
PhoP SF             MRVLVVEDNALLRHHLKVQLQELGHQVDAAEDAREADYYLGEHLPDIAIVDLGLPDEDGL
PhoP SK             MRVLVVEDNALLRHHLKVQLQELGHQVDAAEDAREADYYLGEHLPDIAIVDLGLPDEDGL
PhoP SP             MRVLVVEDNALLRHHLKVQLQELGHQVDAAEDAREADYYLGEHLPDIAIVDLGLPDEDGL
PhoP HM             MRVLVVEDNALLRHHLKVQLQELGHQVDAAEDAREADYYLGEHLPDIAIVDLGLPDEDGL
                    ************************************************************

PhoP KPE16          SLIRRWRSHDVSLPVLVLTAREGWQDKVEVLSAGADDYVTKPFHIEEVAARMQALLRRNS
PhoP MGH78578       SLIRRWRSHDVSLPVLVLTAREGWQDKVEVLSAGADDYVTKPFHIEEVAARMQALLRRNS
PhoP SF             SLIRRWRSHDVSLPVLVLTAREGWQDKVEVLSAGADDYVTKPFHIEEVAARMQALLRRNS
PhoP SK             SLIRRWRSHDVSLPVLVLTAREGWQDKVEVLSAGADDYVTKPFHIEEVAARMQALLRRNS
PhoP SP             SLIRRWRSHDVSLPVLVLTAREGWQDKVEVLSAGADDYVTKPFHIEEVAARMQALLRRNS
PhoP HM             SLIRRWRSHDVSLPVLVLTAREGWQDKVEVLSAGADDYVTKPFHIEEVAARMQALLRRNS
                    ************************************************************

PhoP KPE16          GLASQVISLPPFQVDLSRRELSVNDQPIKLTAFEYTIMETLIRNRGKVVSKDSLMLQLYP
PhoP MGH78578       GLASQVISLPPFQVDLSRRELSVNDQPIKLTAFEYTIMETLIRNRGKVVSKDSLMLQLYP
PhoP SF             GLASQVISLPPFQVDLSRRELSVNDQPIKLTAFEYTIMETLIRNRGKVVSKDSLMLQLYP
PhoP SK             GLASQVISLPPFQVDLSRRELSVNDQPIKLTAFEYTIMETLIRNRGKVVSKDSLMLQLYP
PhoP SP             GLASQVISLPPFQVDLSRRELSVNDQPIKLTAFEYTIMETLIRNRGKVVSKDSLMLQLYP
PhoP HM             GLASQVISLPPFQVDLSRRELSVNDQPIKLTAFEYTIMETLIRNRGKVVSKDSLMLQLYP
                    ************************************************************

PhoP KPE16          DAELRESHTIDVLMGRLRKKIQAEYPQDVITTVRGQGYLFELR
PhoP MGH78578       DAELRESHTIDVLMGRLRKKIQAEYPQDVITTVRGQGYLFELR
PhoP SF             DAELRESHTIDVLMGRLRKKIQAEYPQDVITTVRGQGYLFELR
PhoP SK             DAELRESHTIDVLMGRLRKKIQAEYPQDVITTVRGQGYLFELR
PhoP SP             DAELRESHTIDVLMGRLRKKIQAEYPQDVITTVRGQGYLFELR
PhoP HM             DAELRESHTIDVLMGRLRKKIQAEYPQDVITTVRGQGYLFELR
                    *******************************************
```

(B) **PhoQ**

```
PhoQ MGH78578       MKGLLRHIFPLSLRVRFLLATAGVVLVLSLAYGMVALVGYSVSFDKTTFRLLRGESNLFY
PhoQ KPE16          MKGLLRHIFPLSLRVRFLLATAGVVLVLSLAYGMVALVGYSVSFDKTTFRLLRGESNLFY
PhoQ SF             MKGLLRHIFPLSLRVRFLLATAGVVLVLSLAYGMVALVGYSVSFDKTTFRLLRGESNLFY
PhoQ SK             MKGLLRHIFPLSLRVRFLLATAGVVLVLSLAYGMVALVGYSVSFDKTTFRLLRGESNLFY
PhoQ HM             MKGLLRHIFPLSLRVRFLLATAGVVLVLSLAYGMVALVGYSVSFDKTTFRLLRGESNLFY
PhoQ SP             MKGLLRHIFPLSLRVRFLLATAGVVLVLSLAYGMVALVGYSVSFDKTTFRLLRGESNLFY
                    ************************************************************

PhoQ MGH78578       MLARWENGAIDVDIPENLNMESPTVTLIYDEQGKLLWAQRDVPWLAKRIQPEWLKRNGFH
PhoQ KPE16          MLARWENGAIDVDIPENLNMESPTVTLIYDEQGKLLWAQRDVPWLAKRIQPEWLKRNGFH
PhoQ SF             MLARWENGAIDVDIPENLNMESPTVTLIYDEQGKLLWAQRDVPWLAKRIQPEWLKRNGFH
PhoQ SK             MLARWENGAIDVDIPENLNMESPTVTLIYDEQGKLLWAQRDVPWLAKRIQPEWLKRNGFH
PhoQ HM             MLARWENGAIDVDIPENLNMESPTVTLIYDEQGKLLWAQRDVPWLAKRIQPEWLKRNGFH
PhoQ SP             MLARWENGAIDVDIPENLNMESPTVTLIYDEQGKLLWAQRDVPWLAKRIQPEWLKRNGFH
                    ************************************************************

PhoQ MGH78578       EIEADVDSSSMLLRNNHEIQEQLDAIREQGDDSEMTHSVAINLYPATSKMPQLSIVVVDT
PhoQ KPE16          EIEADVDSSSMLLRNNHEIQEQLDAIREQGDDSEMTHSVAINLYPATSKMPQLSIVVVDT
PhoQ SF             EIEADVDSSSMLLRNNHEIQEQLDAIREQGDDSEMTHSVAINLYPATSKMPQLSIVVVDT
PhoQ SK             EIEADVDSSSMLLRNNHEIQEQLDAIREQGDDSEMTHSVAINLYPATSKMPQLSIVVVDT
PhoQ HM             EIEADVDSSSMLLRNNHEIQEQLDAIREQGDDSEMTHSVAINLYPATSKMPQLSIVVVDT
PhoQ SP             EIEADVDSSSMLLRNNHEIQEQLDAIREQGDDSEMTHSVAINLYPATSKMPQLSIVVVDT
                    ************************************************************

PhoQ MGH78578       IPVELKRSYMVWSWFVYVLAANLLLVIPLLWVAAWWSLRPIESLAKEVRELEEHHREKLN
PhoQ KPE16          IPVELKRSYMVWSWFVYVLAANLLLVIPLLWVAAWWSLRPIESLAKEVRELEEHHREKLN
PhoQ SF             IPVELKRSYMVWSWFVYVLAANLLLVIPLLWVAAWWSLRPIESLAKEVRELEEHHREKLN
PhoQ SK             IPVELKRSYMVWSWFVYVLAANLLLVIPLLWVAAWWSLRPIESLAKEVRELEEHHREKLN
PhoQ HM             IPVELKRSYMVWSWFVYVLAANLLLVIPLLWVAAWWSLRPIESLAKEVRELEEHHREKLN
PhoQ SP             IPVELKRSYMVWSWFVYVLVANLLLVIPLLWVAAWWSLRPIESLAKEVRELEEHHREKLN
                    *******************.****************************************

PhoQ MGH78578       PNTTRELTRLVSNLNRLVRSERERYDKYRTTLTDLTHSLKTPLAVMQSTLRSLRGEKISV
```

```
PhoQ KPE16        PNTTRELTRLVSNLNRLVRSERERYDKYRTTLTDLTHSLKTPLAVMQSTLRSLRGEKISV
PhoQ SF           PNTTRELTRLVSNLNRLVRSERERYDKYRTTLTDLTHSLKTPLAVMQSTLRSLRGEKISV
PhoQ SK           PNTTRELTRLVSNLNRLVRSERERYDKYRTTLTDLTHSLKTPLAVMQSTLRSLRGEKISV
PhoQ HM           PNTTRELTRLVSNLNRLVRSERERYDKYRTTLTDLTHSLKTPLAVMQSTLRSLRGEKISV
PhoQ SP           PNTTRELTRLVSNLNRLVRSERERYDKYRTTLTDLTHSLKTPLAVMQSTLRSLRGEKISV
                  ************************************************************

PhoQ MGH78578     DEAEPVMLEQISRISQQIGYYLHRASMRSGGTLLSRELHPIAPLLDSLTSALNKVYQRKG
PhoQ KPE16        DEAEPVMLEQISRISQQIGYYLHRASMRSGGTLLSRELHPIAPLLDSLTSALNKVYQRKG
PhoQ SF           DEAEPVMLEQISRISQQIGYYLHRASMRSGGTLLSRELHPIAPLLDSLTSALNKVYQRKG
PhoQ SK           DEAEPVMLEQISRISQQIGYYLHRASMRSGGTLLSRELHPIAPLLDSLTSALNKVYQRKG
PhoQ HM           DEAEPVMLEQISRISQQIGYYLHRASMRSGGTLLSRELHPIAPLLDSLTSALNKVYQRKG
PhoQ SP           DEAEPVMLEQISRISQQIGYYLHRASMRSGGTLLSRELHPIAPLLDSLTSALNKVYQRKG
                  ************************************************************

PhoQ MGH78578     VNISLDISPEITFVGEQNDFMEVMGNVLDNACKYCLEFVEVSVRQTTDSHLHILVEDDGP
PhoQ KPE16        VNISLDISPEITFVGEQNDFMEVMGNVLDNACKYCLEFVEVSVRQTTDSHLHILVEDDGP
PhoQ SF           VNISLDISPEITFVGEQNDFMEVMGNVLDNACKYCLEFVEVSVRQTTDSHLHILVEDDGP
PhoQ SK           VNISLDISPEITFVGEQNDFMEVMGNVLDNACKYCLEFVEVSVRQTTDSHLHILVEDDGP
PhoQ HM           VNISLDISPEITFVGEQNDFMEVMGNVLDNACKYCLEFVEVSVRQTTDSHLHILVEDDGP
PhoQ SP           VNISLDISPEITFVGEQNDFMEVMGNVLDNACKYCLEFVEVSVRQTTDSHLHILVEDDGP
                  ************************************************************

PhoQ MGH78578     GIPQSQRRAVFDRGQRADTLRPGQGVGLSVAREIVEQYDGEIIAGESLLGGACMEVVFGR
PhoQ KPE16        GIPQSQRRAVFDRGQRADTLRPGQGVGLSVAREIVEQYDGEIIAGESLLGGACMEVVFGR
PhoQ SF           GIPQSQRRAVFDRGQRADTLRPGQGVGLSVAREIVEQYDGEIIAGESLLGGACMEVVFGR
PhoQ SK           GIPQSQRRAVFDRGQRADTLRPGQGVGLSVAREIVEQYDGEIIAGESLLGGACMEVVFGR
PhoQ HM           GIPQSQRRAVFDRGQRADTLRPGQGVGLSVAREIVEQYDGEIIAGESLLGGACMEVVFGR
PhoQ SP           GIPQSQRRAVFDRGQRADTLRPGQGVGLSVAREIVEQYDGEIIAGESLLGGACMEVVFGR
                  ************************************************************

PhoQ MGH78578     QQMEDKQS
PhoQ KPE16        QQMEDKQS
PhoQ SF           QQMEDKQS
PhoQ SK           QQMEDKQS
PhoQ HM           QQMEDKQS
PhoQ SP           QQMEDKQS
                  ********
```

(C) **PmrA**

```
PmrA MGH78578     MKILVIEDDALLLQGLILAMQSEGYVCDGVSTAHEAALSLASNHYSLIVLDLGLPDEDGL
PmrA SP           MKILVIEDDALLLQGLILAMQSEGYVCDGVSTAHEAALSLASNHYSLIVLDLGLPDEDGL
PmrA SK           MKILVIEDDALLLQGLILAMQSEGYVCDGVSTAHEAALSLASNHYSLIVLDLGLPDEDGL
PmrA HM           MKILVIEDDALLLQGLILAMQSEGYVCDGVSTAHEAALSLASNHYSLIVLDLGLPDEDGL
PmrA SF           MKILVIEDDALLLQGLILAMQSEGYVCDGVSTAHEAALSLASNHYSLIVLDLGLPDEDGL
PmrA KPE16        MKILVIEDDALLLQGLILAMQSEGYVCDGVSTAHEAALSLASNHYSLIVLDLGLPDEDGL
                  ************************************************************

PmrA MGH78578     HFLSRMRREKMTQPVLILTARDTLEDRISGLDTGADDYLVKPFALEELNARIRALLRRHN
PmrA SP           HFLSRMRREKMTQPVLILTARDTLEDRISGLDTGADDYLVKPFALEELNARIRALLRRHN
PmrA SK           HFLSRMRREKMTQPVLILTARDTLEDRISGLDTGADDYLVKPFALEELNARIRALLRRHN
PmrA HM           HFLSRMRREKMTQPVLILTARDTLEDRISGLDTGADDYLVKPFALEELNARIRALLRRHN
PmrA_SF           HFLSRMRREKMTQPVLILTARDTLEDRISGLDTGADDYLVKPFALEELNARIRALLRRHN
PmrA KPE16        HFLTRMRREKMTQPVLILTARDTLEDRISGLDTGADDYLVKPFALEELNARIRALLRRHN
                  ***:********************************************************

PmrA MGH78578     NQGDNEISVGNLRLNVTRRLVWLGETALDLTPKEYALLSRLMMKAGSPVHREILYNDIYS
PmrA SP           NQGDNEISVGNLRLNVTRRLVWLGETALDLTPKEYALLSRLMMKAGSPVHREILYNDIYS
PmrA SK           NQGDNEISVGNLRLNVTRRLVWLGETALDLTPKEYALLSRLMMKAGSPVHREILYNDIYS
PmrA HM           NQGDNEISVGNLRLNVTRRLVWLGETALDLTPKEYALLSRLMMKAGSPVHREILYNDIYS
PmrA SF           NQGDNEISVGNLRLNVTRRLVWLGETALDLTPKEYALLSRLMMKAGSPVHREILYNDIYS
PmrA KPE16        NQGDNEISVGNLRLNVTRRLVWLGETALDLTPKEYALLSRLMMKAGSPVHREILYNDIYS
                  ************************************************************

PmrA MGH78578     WDNEPATNTLEVHIHNLREKIGKSRIRTVRGFGYMLANNIDTE
PmrA SP           WDNEPATNTLEVHIHNLREKIGKSRIRTVRGFGYMLANNIDTE
PmrA SK           WDNEPATNTLEVHIHNLREKIGKSRIRTVRGFGYMLVNNIDTE
PmrA HM           WDNEPATNTLEVHIHNLREKIGKSRIRTVRGFGYMLVNNIDTE
PmrA SF           WDNEPATNTLEVHIHNLREKIGKSRIRTVRGFGYMLVNNIDTE
PmrA KPE16        WDNEPATNTLEVHIHNLREKIGKSRIRTVRGFGYMLANNIDTE
                  ************************************.******
```

(D) **PmrB**

```
PmrB KPE16        MALFATETWTMRHRLLLTIGAILVVCQLISVFWLWHESKEQIQLLVASAIEGHNNQKHVE
PmrB SP           MALFATETWTMRHRLLLTIGAILVVCQLISVFWLWHESKEQIQLLVASAIEGHNNQKHVE
PmrB HM           MALFATETWTMRHRLLLTIGAILVVCQLISVFWLWHESKEQIQLLVASAIEGHNNQKHVE
PmrB MGH78578     MALFATETWTMRHRLLLTIGAILVVCQLISVFWLWHESKEQIQLLVASAIEGHNNQKHVE
PmrB SF           MALFATETWTMRHRLLLTIGAILVVCQLISVFWLWHESKEQIQLLVASAIEGHNNQKHVE
PmrB SK           MALFATETWTMRHRLLLTIGAILVVCQLISVFWLWHESKEQIQLLVASAIEGHNNQKHVE
                  ************************************************************

PmrB KPE16        HEVREAVASLLVPSLLIVGLALYISMLAVRKITRPLSRLQSELENRTPDNLTPIVLSESV
PmrB SP           HEVREAVASLLVPSLLIVGLALYISMLAVRKITRPLSRLQSELENRTPDNLTPIVLSESV
PmrB HM           HEVREAVASLLVPSLLIVGLALYISMLAVRKITRPLSRLQSELENRTPDNLTPIVLSESV
PmrB MGH78578     HEVREAVASLLVPSLLIVGLALYISMLAVRKITRPLSRLQSELENRTPDNLTPIVLSESV
PmrB SF           HEVREAVASLLVPSLLIVGLALYISMLAVRKITRPLSRLQSELENRTPDNLTPIVLSESV
PmrB SK           HEVREAVASLLVPSLLIVGLALYISMLAVRKITRPLSRLQSELENRTPDNLTPIVLSESV
                  ************************************************************

PmrB KPE16        PEVTAVTTALNQLVSRLNLTLDRERLFTADVAHELRTPLAGLRLHLELLSKVHGMGVDPL
PmrB SP           PEVTAVTTALNQLVSRLNLTLDRERLFTADVAHELRTPLAGLRLHLELLAKVHGMGVDPL
PmrB HM           PEVTAVTTALNQLVSRLNLTLDRERLFTADVAHELRTPLAGLRLHLELLAKVHGMGVDPL
PmrB MGH78578     PEVTAVTTALNQLVSRLNLTLDRERLFTADVAHELRTPLAGLRLHLELLAKVHGMGVDPL
PmrB SF           PEVTAVTTALNQLVSRLNLTLDRERLFTADVAHELRTPLAGLRLHLELLAKVHGMGVDPL
PmrB SK           PEVTAVTTALNQLVSRLNLTLDRERLFTADVAHELRTPLAGLRLHLELLAKVHGMGVDPL
                  *************************************************:**********

PmrB KPE16        IQRLDQMTTSISQLLQLARVGQSFSAGSYQQVLLLDDVVKPLQDELEAMLAQRQQRLLLT
PmrB SP           IQRLDQMTTSISQLLQLARVGQSFSAGSYQQVLLLDDVVKPLQDELEAMLAQRQQRLLLT
PmrB HM           IQRLDQMTTSISQLLQLARVGQSFSAGSYQQVLLLDDVVKPLQDELEAMLAQRQQRLLLT
PmrB MGH78578     IQRLDQMTTSISQLLQLARVGQSFSAGSYQQVLLLDDVVKPLQDELEAMLAQRQQRLLLT
PmrB SF           IQRLDQMTTSISQLLQLARVGQSFSAGSYQQVLLLDDVVKPLQDELEAMLAQRQQRLLLT
PmrB SK           IQRLDQMTTSISQLLQLARVGQSFSAGSYQQVLLLDDVVKPLQDELEAMLAQRQQRLLLT
                  ************************************************************

PmrB KPE16        DIENEAVVSGDATLIRVILRNLVENAHRYSPEGSTIRVSVKAGLMPVMAVEDEGPGIDEA
PmrB SP           DIENEAVVSGDATLIRVILRNLVENAHRYSPEGSTIRVSVKAGLMPVMAVEDEGPGIDEA
PmrB HM           DIENEAVVSGDATLIRVILRNLVENAHRYSPEGSTIRVSVKAGLMPVMAVEDEGPGIDEA
PmrB MGH78578     DIENETVVSGDATLIRVILRNLVENAHRYSPEGSTIRVSVKAGLMPVMAVEDEGPGIDEA
PmrB SF           DIENEAVVSGDATLIRVILRNLVENAHRYSPEGSTIRVSVKAGLMPVMAVEDEGPGIDEA
PmrB SK           DIENEAVVSGDATLIRVILRNLVENAHRYSPEGSTIRVSVKAGLMPVMAVEDEGPGIDEA
                  *****:******************************************************

PmrB KPE16        KSGELSKAFVRMDSRYGGIGLGLSIVTRIAQLHDAQFFLHNRQPGPGVRAWVLFPQRGGQ
PmrB_SP           KSGELSKAFVRMDSRYGGIGLGLSIVTRIAQLHDAQFFLHNRQPGPGVRAWVLFPQRGGQ
PmrB HM           KSGELSKAFVRMDSRYGGIGLGLSIVTRIAQLHDAQFFLHNRQPGPGVRAWVLFPQRGGQ
PmrB MGH78578     KSGELSKAFVRMDSRYGGIGLGLSIVTRIAQLHDAQFFLHNRQPGPGVRAWVLFPQRGGQ
PmrB SF           KSGELSKAFVRMDSRYGGIGLGLSIVTRIAQLHDAQFFLHNRQPGPGVRAWVLFPQRGGQ
PmrB SK           KSGELSKAFVRMDSRYGGIGLGLSIVTRIAQLHDAQFFLHNRQPGPGVRAWVLFPQRGGQ
                  ************************************************************

PmrB KPE16        NVSTH
PmrB SP           NVSTH
PmrB HM           NVSTH
PmrB MGH78578     NVSTH
PmrB SF           NVSTH
PmrB SK           NVSTH
                  *****
```

(E) **MgrB**

```
MgrB MGH78578     VKKLRWVLLIVIIAGCLLLWTQMLNVMCDQDVQFFSGICTINKFIPW
MgrB SF           VKKLRWVLLIVIIAGCLLLWTQMLNVMCDQDVQFFSGICTINKFIPW
MgrB SK           VKKLRWVLLIVIIAGCLLLWTQMLNVMCDQDVQFFSGICTINKFIPW
MgrB HM           VKKLRWVLLIVIIAGCLLLWTQMLNVMCDQDVQFFSGICTINKFIPW
MgrB KPE16        VKKLRWVLLIVIIAGCLLLWTQMLNVMCDQDVQFFSGICTINKFIPW
MgrB SP           VKKLRWVLLIVIIAGCLLLWTQMLNVMCDQDVQFFSGICTINKFIPW
                  ***********************************************
```

(F) **YciB/ LapB**

```
LapB SK          MLELLFLLLPVAAAYGWYMGRRSAQQSKQDDASRLSRDYVAGVNFLLSNQQDKAVDLFLD
LapB HM          MLELLFLLLPVAAAYGWYMGRRSAQQSKQDDASRLSRDYVAGVNFLLSNQQDKAVDLFLD
LapB KPE16       MLELLFLLLPVAAAYGWYMGRRSAQQSKQDDASRLSRDYVAGVNFLLSNQQDKAVDLFLD
LapB SF          MLELLFLLLPVAAAYGWYMGRRSAQQSKQDDASRLSRDYVAGVNFLLSNQQDKAVDLFLD
YciM MGH78578    MLELLFLLLPVAAAYGWYMGRRSAQQSKQDDASRLSRDYVAGVNFLLSNQQDKAVDLFLD
LapB SP          MLELLFLLLPVAAAYGWYMGRRSAQQSKQDDASRLSRDYVAGVNFLLSNQQDKAVDLFLD
                 ************************************************************

LapB SK          MLKEDTGTVEAHLTLGNLFRSRGEVDRAIRIHQSLMESASLTYDQRLLAVQQLGRDYMAA
LapB HM          MLKEDTGTVEAHLTLGNLFRSRGEVDRAIRIHQSLMESASLTYDQRLLAVQQLGRDYMAA
LapB KPE16       MLKEDTGTVEAHLTLGNLFRSRGEVDRAIRIHQSLMESASLTYDQRLLAVQQLGRDYMAA
LapB SF          MLKEDTGTVEAHLTLGNLFRSRGEVDRAIRIHQSLMESASLTYDQRLLAVQQLGRDYMAA
YciM MGH78578    MLKEDTGTVEAHLTLGNLFRSRGEVDRAIRIHQSLMESASLTYDQRLLAVQQLGRDYMAA
LapB SP          MLKEDTGTVEAHLTLGNLFRSRGEVDRAIRIHQSLMESASLTYDQRLLAVQQLGRDYMAA
                 ************************************************************

LapB SK          GLYDRAEDMFKQLVDETDFRLGALQQLLQIYQATSDWQSAIEVAERLVKLGKEKHRGEIA
LapB HM          GLYDRAEDMFKQLVDETDFRLGALQQLLQIYQATSDWQSAIEVAERLVKLGKEKHRGEIA
LapB KPE16       GLYDRAEDMFKQLVDETDFRLGALQQLLQIYQATSDWQSAIEVAERLVKLGKEKHRGEIA
LapB SF          GLYDRAEDMFKQLVDETDFRLGALQQLLQIYQATSDWQSAIEVAERLVKLGKEKHRGEIA
YciM MGH78578    GLYDRAEDMFKQLVDETDFRLGALQQLLQIYQATSDWQSAIEVAERLVKLGKEKHRGEIA
LapB SP          GLYDRAEDMFKQLVDETDFRLGALQQLLQIYQATSDWQSAIEVAERLVKLGKEKHRGEIA
                 ************************************************************

LapB SK          NFWCELALQQMAANDLDKAMALLRKGAAADRNSARVSIMMGRVWMEKGDYAKAVESLERV
LapB HM          NFWCELALQQMAANDLDKAMALLRKGAAADRNSARVSIMMGRVWMEKGDYAKAVESLERV
LapB KPE16       NFWCELALQQMAANDLDKAMALLRKGAAADRNSARVSIMMGRVWMEKGDYAKAVESLERV
LapB SF          NFWCELALQQMAANDLDKAMALLRKGAAADRNSARVSIMMGRVWMEKGDYAKAVESLERV
YciM MGH78578    NFWCELALQQMAANDLDKAMALLRKGAAADRNSARVSIMMGRVWMEKGDYAKAVESLERV
LapB SP          NFWCELALQQMAANDLDKAMALLRKGAAADRTSARVSIMMGRVWMEKGDYAKAVESLERV
                 *******************************.****************************

LapB SK          IDQDKELVGETLEMLQTCYQQLGKTDEWEVFLRRCVEENAGATAELMLAQILEQREGVEA
LapB HM          IDQDKELVGETLEMLQTCYQQLGKTDEWEVFLRRCVEENAGATAELMLAQILEQREGVEA
LapB KPE16       IDQDKELVGETLEMLQTCYQQLGKTDEWEVFLRRCVEENAGATAELMLAQILEQREGVEA
LapB SF          IDQDKELVGETLEMLQTCYQQLGKTDEWEVFLRRCVEENAGATAELMLAQILEQREGVEA
YciM MGH78578    IDQDKELVGETLEMLQTCYQQLGKTDEWEVFLRRCVEENAGATAELMLAQILEQREGVEA
LapB SP          IDQDKELVGETLEMLQTCYQQLGKTDEWEVFLRRCVEENAGATAELMLAQILEQREGVEA
                 ************************************************************

LapB SK          AQNYVTRQLERHPTMRVFHKLMDYHLNEAEEGRAKESLGVLRNMVGEQVRSKPRYRCQKC
LapB HM          AQNYVTRQLERHPTMRVFHKLMDYHLNEAEEGRAKESLGVLRNMVGEQVRSKPRYRCQKC
LapB KPE16       AQNYVTRQLERHPTMRVFHKLMDYHLNEAEEGRAKESLGVLRNMVGEQVRSKPRYRCQKC
LapB SF          AQNYVTRQLERHPTMRVFHKLMDYHLNEAEEGRAKESLGVLRNMVGEQVRSKPRYRCQKC
YciM MGH78578    AQNYVTRQLERHPTMRVFHKLMDYHLNEAEEGRAKESLGVLRNMVGEQVRSKPRYRCQKC
LapB SP          AQNYVTRQLERHPTMRVFHKLMDYHLNEAEEGRAKESLGVLRNMVGEQVRSKPRYRCQKC
                 ************************************************************

LapB SK          GFTAHTLYWHCPSCRSWATIKPIRGLDGQ
LapB HM          GFTAHTLYWHCPSCRSWATIKPIRGLDGQ
LapB KPE16       GFTAHTLYWHCPSCRSWATIKPIRGLDGQ
LapB SF          GFTAHTLYWHCPSCRSWATIKPIRGLDGQ
YciM MGH78578    GFTAHTLYWHCPSCRSWATIKPIRGLDGQ
LapB SP          GFTAHTLYWHCPSCRSWATIKPIRGLDGQ
                 *****************************
```

(G) **LpxM/ MsbB**

```
LpxM HM          METKKNNIEFIPKFEKSFLLPRYWGAWLGVFAFAGIALTPPSFRDPLLGKLGRLVGRLAK
LpxM SP          METKKNNIEFIPKFEKSFLLPRYWGAWLGVFAFAGIALTPPSFRDPLLGKLGRLVGRLAK
LpxM SF          METKKNNIEFIPKFEKSFLLPRYWGAWLGVFAFAGIALTPPSFRDPLLGKLGRLVGRLAK
LpxM SK          METKKNNIEFIPKFEKSFLLPRYWGAWLGVFAFAGIALTPPSFRDPLLGKLGRLVGRLAK
MsbB MGH78578    METKKNNIEFIPKFEKSFLLPRYWGAWLGVFAFAGIALTPPSFRDPLLGKLGRLVGRLAK
LpxM KPE16       METKKNNIEFIPKFEKSFLLPRYWGAWLGVFAFAGIALTPPSFRDPLLGKLGRLVGRLAK
                 ************************************************************
```

```
LpxM HM          SSRRRAQINLLYCFPEKSEYEREAIIDAMYASAPQAMVMMAELGLRDPQKILARVDWQGK
LpxM SP          SSRRRAQINLLYCFPEKSEYEREAIIDAMYASAPQAMVMMAELGLRDPQKILARVDWQGK
LpxM SF          SSRRRAQINLLYCFPEKSEYEREAIIDAMYASAPQAMVMMAELGLRDPQKILARVDWQGK
LpxM SK          SSRRRAQINLLYCFPEKSEYEREAIIDAMYASAPQAMVMMAELGLRDPQKILARVDWQGK
MsbB MGH78578    SSRRRAQINLLYCFPEKSEYEREAIIDAMYASAPQAMVMMAELGLRDPQKILARVDWQGK
LpxM KPE16       SSRRRAQINLLYCFPEKSEYEREAIIDAMYASAPQAMVMMAELGLRDPQKILARVDWQGK
                 ************************************************************

LpxM HM          AIIDEMQRNNEKVIFLVPHAWGVDIPAMLMASGGQKMAAMFHNQGNPVFDYVWNTVRRRF
LpxM SP          AIIDEMQRNNEKVIFLVPHAWGVDIPAMLMASGGQKMAAMFHNQGNPVFDYVWNTVRRRF
LpxM SF          AIIDEMQRNNEKVIFLVPHAWGVDIPAMLMASGGQKMAAMFHNQGNPVFDYVWNTVRRRF
LpxM SK          AIIDEMQRNNEKVIFLVPHAWGVDIPAMLMASGGQKMAAMFHNQGNPVFDYVWNTVRRRF
MsbB MGH78578    AIIDEMQRNNEKVIFLVPHAWGVDIPAMLMASGGQKMAAMFHNQGNPVFDYVWNTVRRRF
LpxM KPE16       AIIDEMQRNNEKVIFLVPHAWGVDIPAMLMASGGQKMAAMFHNQGNPVFDYVWNTVRRRF
                 ************************************************************

LpxM HM          GGRMHARNDGIKPFIQSVRQGYWGYYLPDQDHGAEHSEFVDFFATYKATLPAIGRLMKVC
LpxM SP          GGRMHARNDGIKPFIQSVRQGYWGYYLPDQDHGAEHSEFVDFFATYKATLPAIGRLMKVC
LpxM SF          GGRMHARNDGIKPFIQSVRQGYWGYYLPDQDHGAEHSEFVDFFATYKATLPAIGRLMKVC
LpxM SK          GGRMHARNDGIKPFIQSVRQGYWGYYLPDQDHGAEHSEFVDFFATYKATLPAIGRLMKVC
MsbB MGH78578    GGRMHARNDGIKPFIQSVRQGYWGYYLPDQDHGAEHSEFVDFFATYKATLPAIGRLMKVC
LpxM KPE16       GGRMHARNDGIKPFIQSVRQGYWGYYLPDQDHGAEHSEFVDFFATYKATLPAIGRLMKVC
                 ************************************************************

LpxM HM          RARVVPLFPVYDGKTHRLTVLVRPPMDDLLDADDTTIARRMNEEVEVFVKPHTEQYTWIL
LpxM SP          RARVVPLFPVYDGKTHRLTVLVRPPMDDLLDADDTTIARRMNEEVEVFVKPHTEQYTWIL
LpxM SF          RARVVPLFPVYDGKTHRLTVLVRPPMDDLLDADDTTIARRMNEEVEVFVKPHTEQYTWIL
LpxM SK          RARVVPLFPVYDGKTHRLTVLVRPPMDDLLDADDTTIARRMNEEVEVFVKPHTEQYTWIL
MsbB MGH78578    RARVVPLFPVYDSKTHRLTVLVRPPMDDLLDADDTTIARRMNEEVEVFVKPHTEQYTWIL
LpxM KPE16       RARVVPLFPVYDSKTHRLTVLVRPPMDDLLDADDTTIARRMNEEVEVFVKPHTEQYTWIL
                 ************.***********************************************

LpxM HM          KLLKTRKPGEIEPYKRKELFPKKK
LpxM SP          KLLKTRKPGEIEPYKRKELFPKKK
LpxM SF          KLLKTRKPGEIEPYKRKELFPKKK
LpxM SK          KLLKTRKPGEIEPYKRKELFPKKK
MsbB MGH78578    KLLKTRKPGEIEPYKRKELFPKKK
LpxM KPE16       KLLKTRKPGEIEPYKRKELFPKKK
                 ************************
```
